# Bridging the research to practice gap: a systematic scoping review of implementation of interventions for cancer-related fatigue management

**DOI:** 10.1186/s12885-021-08394-3

**Published:** 2021-07-14

**Authors:** Oluwaseyifunmi Andi Agbejule, Nicolas H. Hart, Stuart Ekberg, Koczwara Bogda, Rahul Ladwa, Camilla Simonsen, Elizabeth P. Pinkham, Raymond Javan Chan

**Affiliations:** 1 Cancer and Palliative Care Outcomes Centre, School of Nursing, N Block, Kelvin Grove Campus,, Queensland University of Technology (QUT), Kelvin Grove, Queensland 4059 Australia; 2 Exercise Medicine Research Institute, Edith Cowan University, Perth, Western Australia 6027 Australia; 3 Institute for Health Research, University of Notre Dame Australia, Fremantle, Western Australia 6959 Australia; 4 Flinders University and Flinders Medical Centre, Flinders Drive, Bedford Park, South Australia 5048 Australia; 5 Princess Alexandra Hospital, Metro South Hospital and Health Services, Woolloongabba, Queensland 4102 Australia; 6 School of Medicine, University of Queensland, St Lucia, Queensland 4072 Australia; 7 School of Health and Behavioural Science, University of Queensland, St Lucia, Queensland 4072 Australia

**Keywords:** Cancer-related fatigue, Exercise, Implementation science, Oncology, Physical activity, Survivorship

## Abstract

**Background:**

Cancer-related fatigue (CRF) is one of the most common and distressing symptoms in people with cancer. Although efficacy of interventions for CRF have been extensively investigated, less has been done to ensure successful translation into routine clinical practice. The aim of this systematic scoping review was to synthesise knowledge surrounding the implementation of CRF interventions, summarise the processes and outcomes of implementation strategies used, and identify opportunities for further research.

**Methods:**

PubMed, Cochrane CENTRAL, EMBASE and CINAHL databases were searched (up to December 2020). The Cochrane Effective Practice and Organisation of Care (EPOC) Group taxonomy and the RE-AIM Framework were used to guide the evaluation of implementation strategies and outcomes, respectively.

**Results:**

Six studies were included. Three used an implementation framework (PARIHS, KTA, Cullens & Adams’ Implementation Guide) to guide implementation. Overall, the implementation strategies used across all studies were reported to have directly resulted in immediate changes at the clinician level (e.g., increased clinician behaviours, self-efficacy, attitudes, knowledge of CRF management). No clear relationship was found between the use of implementation models and the number or type of implementation strategies used. For outcomes, Effectiveness and Implementation were the most highly reported RE-AIM measures followed by Reach then Maintenance. Adoption was the least reported.

**Conclusions:**

Despite the high prevalence of CRF and evidence-based interventions for managing CRF, there is limited evidence informing the sustainable implementation of these interventions. This systematic scoping review emphasises the lack of quality CRF implementation studies presently available in the literature leading to a disconnect between effective CRF interventions, routine clinical care, and cancer survivors at present. This review highlights the need for robust study designs guided by established frameworks to methodically design and evaluate the implementation of CRF management interventions in the future.

**Supplementary Information:**

The online version contains supplementary material available at 10.1186/s12885-021-08394-3.

## Background

Cancer-related fatigue (CRF) is experienced by over 60% of cancer survivors depending on their cancer diagnosis and associated treatments, with two-thirds reporting severe CRF extending beyond 6 months, and one-third reporting persistent CRF over many years [1]. While CRF is known as one of the most distressing and prevalent symptoms experienced by people with cancer [2, 3], it has no current universal definition, with the National Comprehensive Cancer Network (NCCN) describing it as “a persistent, subjective sense of physical, emotional and/or cognitive exhaustion related to cancer or cancer treatment that is not proportional to recent activity and interferes with usual functioning” [4]. CRF greatly diminishes patients’ physical, mental, occupational, emotional and social wellbeing during and after treatment [2, 5, 6]. Other than reduced quality of life, CRF can also lead to difficulties in decision making, daily living disruption and an increased dependency on others [7]. Such impacts on quality of life (QoL) have been widely reported by a broad range of cancer survivor populations [4, 6–13].

Various interventions for managing CRF have been investigated. These include physical activity and exercise (e.g., aerobic, resistance), pharmacological interventions (e.g., erythropoietin, methylphenidate, modafinil), psychological interventions (e.g., cognitive behaviour therapy), and integrative therapies (e.g., acupuncture, massage). There is level one evidence [14–18] supporting the benefits of physical activity, exercise and psychological interventions for managing CRF. In a recent meta-analysis, Mustian and colleagues [16] identified that exercise (weighted effect size [WES], 0.30; 95% CI, 0.25–0.36; *P* < .001) and psychological interventions (WES, 0.27; 95% CI, 0.21–0.33; *P* < .001) produced significant moderate positive effects on CRF improvement, with authors suggesting that both management strategies be prescribed as first line therapy. A plethora of research has focused on the efficacy of CRF interventions; however, there is much to learn about how these interventions can be incorporated into healthcare and ‘real-world’ settings.

While the discipline of implementation science is gaining momentum, less than half of interventions found to be effective in disease management and prevention are ever adopted into clinical use and routine practice [19–21]. Over recent years, cancer care and health service leaders are increasingly concentrating their efforts on facilitating the systematic uptake of research findings into routine care to improve service and patient outcomes [22]. There are numerous systematic reviews on the clinical efficacy of CRF interventions; however, to our knowledge there is no comprehensive review focussing on the implementation of CRF management interventions and programs. To address this gap, this systematic scoping review of the CRF literature was conducted to answer the following key questions: (1) What current efforts have been made to implement CRF interventions in clinical care?; (2) What implementation frameworks, strategies, theories or models have been used when implementing CRF interventions in clinical care?; and (3) What were the outcomes of identified CRF interventions and implementation efforts?

## Methods

This review sought to examine implementation in CRF literature and identify possible knowledge gaps, thus a scoping methodology was adopted [23]. This systematic scoping review was conducted according to the Preferred Reporting Items for Systematic Reviews and Meta-Analyses Extension for Scoping Reviews (PRISMA-ScR) guidelines [24] (See Additional File 1).

### Eligibility criteria

The population for this review were cancer survivors (regardless of age, gender, tumour and treatment type) at any stage of their cancer trajectory that have experienced fatigue as a result of their cancer or cancer treatment. The taxonomy of implementation strategies developed by the Cochrane Effective Practice and Organisation of Care (EPOC) Group [25] were used to determine the definition and inclusion of implementation studies in the review (see Additional File 2). These EPOC implementation strategies were developed for interventions that targeted and produced changes at the healthcare organisation level, healthcare professional level and the health service level and thus were considered relevant for this review.

For inclusion, studies were required to meet the following criteria: 1) have the implementation of an intervention/program/guideline as a primary goal; 2) have cancer-related fatigue as a primary symptom of interest; 3) incorporate at least one of the EPOC implementation strategies; 4) be published in English; and 5) have full-text available.

No restrictions were placed on types of study designs eligible for inclusion. As the key interest of our systematic scoping review is to describe implementation outcomes of CRF management interventions, we included original research articles (i.e., randomised controlled trials, observational studies, qualitative studies, mixed methods, abstracts, program evaluations) and other grey literature (e.g., evaluations of modules, online programs and institutional/government interventions). Descriptive articles (i.e., commentaries, editorials, recommendation reports/articles) were excluded.

### Search strategy

Four databases (PubMed, Cochrane CENTRAL, EMBASE and CINAHL) were searched (up to December 2020) as outlined in Additional File 3. Free text terms and relevant subject headings (i.e., MeSH, EMTREE) for “cancer-related fatigue” (cancer fatigue, fatigue) and “interventions” were used. These terms were also combined with implementation study terms (e.g., “implementation”, “translation”, “program development”, etc.) using the Boolean logic operators (or, and). Reference and citation lists of relevant articles were also hand searched for eligible studies that met the inclusion criteria. Titles and abstracts of articles retrieved from the search strategy were independently screened by two authors (CS, OAA). The same two authors then assessed the eligibility of relevant full-text articles for inclusion in the review. Disagreements were resolved through consensus among the two authors, with a third author (RC) as arbiter where required.

### Data extraction

Data extraction was conducted by one author (OAA) and checked for accuracy by a second author (RC). Key information extracted included: study characteristics (i.e., author, publication year, study design, purpose, participants, sample size); intervention characteristics (i.e., setting, context, model of care, resources used, intervention description); implementation framework or theory used; implementation strategies used; and implementation outcomes. A model or framework was considered specific to implementation if it described the process of translating research into practice, explained the influences of implementation outcomes, or evaluated implementation processes [26]. Implementation strategies were categorised using the components from the EPOC taxonomy (see Additional File 2).

The RE-AIM framework [27] was used to catalogue the outcomes of strategies, methods or techniques designed to change clinician or patient behaviours related to CRF. RE-AIM was initially developed to balance emphasis on internal and external validity and to expand on assessments of interventions beyond efficacy [28–30]. The RE-AIM dimensions include reach (R), effectiveness/efficacy (E), and maintenance (M)–which operate at the individual-level (i.e., rate or participation, intervention success rate, and endurance of individual behaviour respectively); and adoption (A), implementation (I), and maintenance (M), which focus on the organisation level [31]. In our review, data were extracted using a widely used [28, 30] RE-AIM coding sheet for systematic reviews published on the RE-AIM website (http://www.re-aim.org/resources-and-tools/measures-and-checklists/ - Additional File 4). Due to the heterogeneity of included studies, a narrative synthesis was conducted. As the purpose of this review was to provide an overview of existing evidence regardless of methodological quality or risk of bias, no quality assessment was conducted, consistent with the PRISMA-ScR guidelines.

## Results

**Fig. 1:**
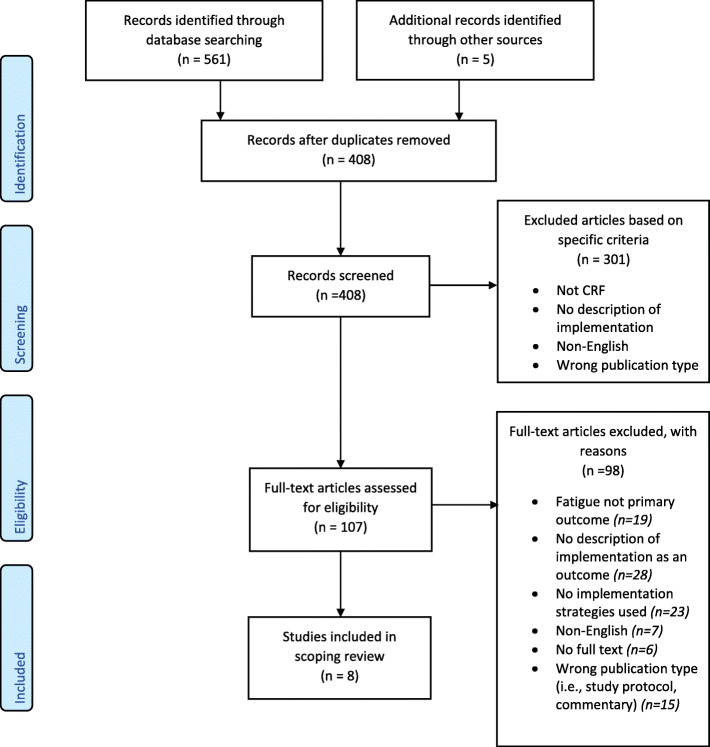
PRISMA Flow Diagram

Database searches resulted in 561 potentially eligible records. Of these, eight articles representing six implementation studies [32–37] met the inclusion criteria and were included in the review (See PRISMA Flow Chart: Fig. 1).

### Characteristics of included studies and programs

Included studies are described in Table 1. Of the six studies included, three were conducted in the USA [34, 36, 37], two in China [32, 35], and one in Canada [33]. Study design varied and consisted of one clinical audit implementation study [32], one qualitative program evaluation [37], three pre-test, post-test single group observation studies [33, 35, 36], and one longitudinal 3-group quasi-experimental comparative study [34]. Three studies [32, 33, 35] examined the impact of implementation efforts on the adoption of CRF evidenced-based guidelines, of which two [32, 33] reported the impact of implementation strategies at the organisational and health professional level, and one [35] described the impact of implementation efforts on oncology nurses and patients. The remaining three studies [34, 36, 37] investigated the efficacy of CRF interventions implemented at the patient level. Participants in each of the three efficacy studies [34, 36, 37] had mixed tumour types (i.e., breast, colon, lung, gastrointestinal, prostate, ovarian, uterine, myeloma, non-Hodgkin and Hodgkin lymphoma cancers). CRF interventions described in these three studies were physical activity and exercise [36], clinician and patient education [34], or combined exercise and education [37]. Of the two studies with education components [34, 37], only ‘information giving’ education strategies were described (e.g., information sessions, printed material). Types of physical activity described were aerobic exercise (e.g., walking, treadmill, Nu-Step), resistance training (e.g., resistance bands), balance and stretching, yoga and aquatic exercises [36, 37]. Of the three studies that implemented CRF interventions [34, 36, 37], two [34, 36] reported the impact of CRF interventions on patient fatigue outcomes (e.g., reduced fatigue).

**Table 1:** Summary of Included Articles

Author, year [Program name], country	Study design /Setting/ Sample Size	Purpose	Participants	Models of Care	Resources Used/ Described	Intervention/ Program Description	Implementation Model/ Framework/ Theories
Huether et al., 2016 [36] / Energy Through Motion©]/Iowa United States	Pre-, post-test single group/ Ambulatory / (*n* = 39) Pre-, post-test single group / 2 Survivorship clinics/ (*n* = 50)	Feasibility (Abbott et al., 2017) Effectiveness (Huether et al., 2016)	Adult Cancer Survivors	Nurse-led survivorship clinics Home-based exercise	Activity trackers, Resistance Bands, Verbal instruction, Printed material (i.e., exercise & calorie guides), Pedometers, Activity logs, Bottle	3-month physical activity program that incorporated education, a specialised kit (including info on PA benefits, exercise equipment, sleep strategies logbook, home workouts) and ongoing patient support. The program also included an intricate text message system provide information and encouragement and promote adherence.	(Cullen & Adams, 2002) Implementation Strategies for Best Practice Guide
**EPOC Implementation Strategies** System/health professional level **Reminders:** update practice reminders provided by project leader to regularly reinforce the program. **Local Opinion leaders**: DNP student, clinical nurse specialist, nurse practitioners informed content and development. **Continuous Quality Improvement:** Senior leaders, existing and new team members were regularly updated on intervention progress. ● Dedicated quality improvement program. ● Protocol revisions based on feedback from clinicians, patients, or family. **Managerial supervision & Monitoring Performance**: Regular reports to senior leaders. **Interprofessional Education & Educational meetings:** Presentations on evidence on CRF, physical activity and the Energy Through Motion program at staff meetings, unit in-services, **Educational materials:** pocket guides provided for clinicians					**Outcomes** Effectiveness • Results **° Fatigue**: Decreased by an average of 2 points compared to an increase of 0.69 in usual care arm (p = 0.0006) **° Activity Levels:** Increased activity levels by a mean of 2.59 points (*p* = 0.0016) compared to usual care (decreased levels by a mean of 1.07) **° QOL:** Improved in all measured areas from 1.24–2.41 points (0–10 scale) compared to decrease (0.69–1.14 points) in usual care. **° Program evaluation:** Participants reported that the program was helpful and beneficial. Connecting activity trackers to the computer and follow-along activity videos considered least helpful (Information videos also reported as not used regularly). • Percent attrition: 10% Implementation • **Completion rate:** 90% Maintenance (Setting Level) Program still maintained at the University of Iowa Holden Comprehensive Cancer Centre		
**Author, year [Program name], country**	**Study design /Setting/ Sample Size**	**Purpose**	**Participants**	**Models of Care**	**Resources Used/ Described**	**Intervention/ Program Description**	**Implementation Model/ Framework/ Theories**
Borneman et al., 2011 [34]/ [Passport to Comfort]/ California, United States	Quasi-experimental comparative study/Ambulatory/ (Phase 1 [usual care] *n* = 83, Phase 2 [intervention] *n* = 104, Phase 3 [Dissemination] *n* = 93)	Phase 1 &2: Effectiveness Phase 3: Dissemination	Patients with breast, colon, lung and prostate cancer (at least 1 month after diagnosis).	Nurse-led	Teaching Packet consisting of written educational materials.	Phase 1, 2: Psycho-educational intervention. Each patient received 4 (approx. 60 min) educational sessions as well as written information material or ‘tip’ sheets in a ‘teaching packet’. Tip sheets provided education on exercise, nutrition, emotional issues and sleep disturbance. During sessions, information on pain assessment, fatigue assessment and fatigue management was provided. A month after last educational session participants received bi-weekly follow-up phone calls every 2 weeks for 3 months.	None Described
**EPOC Implementation Strategies:** System/ Health Professional Level **Local Consensus Processes & Clinical Guidelines:** Intervention informed by the National Comprehensive Cancer Network (NCCN). A ‘Patient Pain Knowledge Tool’ was created based on NCCN pain guidelines. **Educational Meetings & Material:** Regular meetings with nurses. ● Pain and fatigue presentations by national experts to oncologists and nurse practitioners. ● Monthly newsletter to practitioners for ongoing education and communication. ● Internal Advisory Board met quarterly to gain clinician input from researchers involved in the intervention. **Organisational Culture (Phase 3):** Pain and fatigue education provided to all clinicians at a total of 38 in-services. ● Pain and fatigue information provided at key meetings. ● Routine fatigue assessment added to outpatient clinic vital sign flow sheet. ● Increased referrals to supportive care departments for pain and fatigue. ● Patient education materials were translated into Spanish. ● Patient education materials made available on employee Intranet. ● Advocacy posters placed around clinic to remind staff and patients to discuss fatigue. ● **Audit and feedback:** Clinical feedback reports completed for patients and provided to MDs and NPs based on chart audits with specific feedback for pain and fatigue management. **Tailored interventions:** Strategies created to address identified patient, professional and system barriers.					**Outcomes** Reach • **Participation rate** (Phase 3): 93% Effectiveness • Phase 1 & 2: **Fatigue -** fatigue management barriers were significantly higher in the usual care group than in the intervention group. The usual care group had significantly more fatigue (beta = −0.155). • Phase 3: **Fatigue -** Significant immediate and sustained effects were shown on the Fatigue Barriers Scale (FBS) for the intervention group. The intervention group demonstrated a significant delayed effect in Physical QOL – maintained baseline levels of QOL throughout the study when we would normally expect a decrease in QOL. Statistically significant differences between QOL measures were small. Maintenance (Individual) • (2-month follow-up) Attrition rate: 32.5%		
**Author, year [Program name], country**	**Study design /Setting/ Sample Size**	**Purpose**	**Participants**	**Models of Care**	**Resources Used/ Described**	**Intervention/ Program Description**	**Implementation Model/ Framework/ Theories**
Jones et al., 2020 [33]/ Canada	Prospective Cohort/(*n* = 18)	Implementation Intervention Acceptability and Feasibility	Healthcare professional and community support workers	N/A	Flipchart/Checklist– summarised guidelines, screening, and assessment information.	A one-time in person 2-h training session offered to health care practitioners and community support providers about the CAPO CRF guidelines. First hour provided information on practice gaps reported in literature, CAPO CRF guidelines, communication skills and motivational interviewing principles. Second hour involved role-play and group discussions.	Knowledge to Action (KTA) Model
**EPOC Implementation Strategies** **Clinical Guidelines:** Canadian Association of Psychosocial Oncology (CAPO) guidelines for CRF used to inform education sessions. **Local Consensus Processes:** Clinical guidelines adapted to the Ottawa context after consensus amongst stakeholders. **Local opinion leaders & Tailored Intervention:** Focus interviews and program development with stakeholder groups (patients, health care professionals and community support professionals, pedagogy expert) to identify barriers to change; subsequent strategies then created. **Educational materials**: All participants provided with a flipchart that contains information on assessing and managing fatigue.					**Outcomes** Reach **Participation Rate**: 90% Implementation Impact • Program was effective in increasing knowledge, self-efficacy and intent to apply guidelines. • **CRF Knowledge:** −3.959(14), p = 0.001) with a large effect size (d = 0.98). • **Self-Efficacy in CRF Assessment** (t = 2.621(13), *p* = 0.021) with a large effect size (d = 0.88). • **Self-efficacy to intervene for CRF** (t = 2.924(13), *p* = 0.012) with a large effect size (d = 1.13). • **Intent to apply Clinical Guidelines in Practice:** t = 4.786(13), *p* = 0.000) with a large effect size (d = 1.35). • **Feasibility:** mean satisfaction score (52.27 ± 6.97 out of 60 points maximum). Implementation **Completion Rate:** 88.9%		
**Author, year [Program name], country**	**Study design /Setting/ Sample Size**	**Purpose**	**Participants**	**Models of Care**	**Resources Used/ Described**	**Intervention/ Program Description**	**Implementation Model/ Framework/ Theories**
Tian et al. 2017 [35]/ China	Pre-test, post-test/ Radiotherapy Unit & Medical Oncology Unit/N/A	Implementation – Intervention Translating guidelines into practice	Nursing Staff	N/A	Nursing record chart, CRF education booklet, CRF quality control checklist	Study outlined the creation of a ‘CRF Nursing Guideline’ using a steering group (consisting of clinical experts). The resulting guidelines were implemented into practice through an evidenced-based project utilising training and education for nurses, changes to nursing procedures (screening and assessment and quality review) and the provision of staff resources. Impact of the project was measured at the organisational, staff and patient level.	The Promoting Action on Research Implementation in Health Services (PARIHS) framework
**EPOC Implementation Strategies:** System/health professional level **Continuous Quality Improvement & Audit and Feedback:** Feedback and suggestions periodically collected to determine whether further specific training or modification (to nursing procedure) was required. **Educational materials:** CRF Education booklet and other training print materials given to nursing staff. **Educational meetings:** Training courses on CRF nursing care were established, including elementary training on evidence-based nursing practice and specific training on CRF nursing care. Seminars on evidence-based practice concerning CRF management. **Clinical Practice Guidelines/ Local Consensus Processes:** The “Clinical Practice Guideline: Nursing Care of Cancer-Related Fatigue in Adults with Cancer was developed by interventionists. **Local opinion leaders:** Creation of a steering group (six directors from nursing, medical oncology, radiotherapy, Traditional Chinese Medicine, Psychiatry departments. ● Opinion leader identified to change nurse negative opinion of the project, train other nurses and act as a role model for fellow clinicians. ● Integration of existing staff into facilitation team. **Tailored Intervention:** Initial focus groups and discussions conducted to identify barriers to change. Subsequent strategies then created.					**Outcomes** Implementation Impact • Nurse Outcomes: After implementation of the project, knowledge, attitudes, and behaviour scores were all higher than at baseline. • Patient Outcomes: ° No differences were detected between the baseline and final scores of the “self-efficacy questionnaire for CRF management” (SQFM) scale. ° Patients adopted more effective CRF management strategies (previously just rested to alleviate fatigue) ° CRF scores lower after intervention than prior to intervention [5.59(2.09) vs. 6.50 (1.90); t = 2.22, *p* = 0.04].		
**Author, year [Program name], country**	**Study design /Setting/ Sample Size**	**Purpose**	**Participants**	**Models of Care**	**Resources Used/ Described**	**Intervention/ Program Description**	**Implementation Model/ Framework/ Theories**
Van Gerpen & Becker, 2013 [37]/ [LifeSpring]/ United States	Program Evaluation Article /Wellness Centre/N/A	Program Evaluation	Adult Cancer Survivors	Physical therapist and exercise physiologist led	Resistance Bands T-shirts w/LifeSpring logo Written Hand out materials Snacks Balloons (for release at graduation) Exercise equipment (dumbbells, machines)	Exercise and education program. 12-week program consisting of a bi-weekly exercise session and a weekly education session. Exercise component consists of: 20–30-min of aerobic exercise (5-min intervals of stationary bike, treadmill, walking on the indoor track, recumbent stepper (Nu-SteP), or upper-body ergometer) and 20–30-min of group exercises (resistance training, balance/flexibility/stretching exercises, aquatic exercises and relaxation, Pilates or BODYFLOW™ exercises). Educational sessions are led by content experts and include topics such as exercise and cancer, healing, communication and coping, spirituality sleep.	None Described
**EPOC Implementation Strategies:** System/Health Professional Level **Local Opinion leaders:** Program development by physical therapist, medical and radiation oncologists, general surgeons, nurses, cancer survivors. ● Phone interviews with intervention leaders from other programs to provide additional insight on recruitment, retention, program design education session topics, screening tools, etc. **Continuous Quality Improvement:** Program was modified to include all cancer types (originally only breast cancer survivors) after results from initial program evaluation. Sessions were limited to 12 participants after previous larger class sizes caused challenges in providing individualised support.					**Outcomes** Effectiveness/ Efficacy Participants reported improvements in their fatigue, pain, sleep disturbances, depression, and quality of life, (demonstrated from their pre-, mid-, and post program scores). • • Fatigue: [5.58 (2.11) vs. 3.55 (1.86); *p* < 0.0001] • Sleep [4.77 (2.5) vs. 3.26 (2.27); p < 0.0001], • Quality of life [3.63 (2.27) vs. 2.08 (1.86); p < 0.0001], • Pain [2.52 (2.31) vs. 1.85 (1.85); *p* < 0.001], • Depression [2.72 (2.21) vs. 1.65 (1.49); p < 0.0001]. Implementation **Participation/Attendance Rates:** 80%. From 2007 to approx. 2013: 182 participated in program and 152 completed the program Maintenance (Setting Level) Program still maintained at the Bryan Health Medical Centre		
**Author, year [Program name], country**	**Study design /Setting/ Sample Size**	**Purpose**	**Participants**	**Models of Care**	**Resources Used/ Described**	**Intervention/ Program Description**	**Implementation Model/ Framework/ Theories**
Wang et al., 2018 [32]/ China	Clinical Audit/ Hospital/ N/A	Implementation Project	Nursing Staff	N/A	Educational materials	This article first determined the current state of CRF management in the oncology department by undertaking an initial audit. Strategies (listed below) were then implemented to improve practice and address the barriers identified. A follow-up audit was conducted to evaluate the impact of changes made.	None described. Researchers used the JBI Getting Research into Practice (GRiP) tool to identify barriers and practice gaps.
**EPOC Implementation Strategies** **Local Opinion Leaders:** Routine communication with stakeholders to inform strategy development and promote good clinical practice. **Clinical Guidelines:** Content of all educational materials derived from the NCCN CRF guidelines. **Organisational Culture & Educational materials:** Information brochures and posters about CRF management strategies developed for patients and staff. ● Flow chart detailing CRF assessment steps created and displayed in nursing unit. ● Paper-based CRF assessment tools (including BFI) created and distributed for use. **Educational meetings:** Formal 2-h education sessions on CRF background, management and assessment delivered to all nurses. ● ‘Practice fatigue assessments’ and patient education sessions completed by nurses (under supervision). **Monitoring Performance & Managerial Supervision:** Ongoing discussions, communication, and monitoring of nurses to ensure compliance. **Tailored interventions:** Clinical audit conducted to address barriers to change. ● Procedures adjusted to account for changes in environment, workload and time restrictions (i.e., reallocation of work tasks, management support, time management via prioritisation of work tasks, balancing resources).					**Outcomes** Implementation Impact Compliance with best practice audit criteria (compliance rates) - • Health professional received education and training: Baseline audit – **0%** Follow up audit - **97%** • CRF assessment upon admission and at regular intervals throughout care: Baseline audit **– 0%** Follow up audit - **86%** • Focused assessment of fatigue undertaken in patients who screen positively: Baseline audit – 0% Follow up audit - **64%** • Patient education about physical activities: Baseline audit – **3%,** Follow up audit - **78%** • Patient informed about the strategies to manage cancer related fatigue: Baseline audit **– 0%** Follow up audit - **83%**		

*Abbreviations*: *BFI* Brief Fatigue Inventory, *CAPO* Canadian Association for Psychosocial Oncology, *CRF* Cancer-related Fatigue, *DNP* Doctor of Nursing Practice, *JBI* Joanna Briggs Institute, *MD* Doctor of Medicine, *NCCN* National Comprehensive Cancer Network, *N/A* Not applicable, *NP* nurse practitioner, *PA* physical activity, *QOL* Quality of Life

### Implementation models and frameworks

Only three of six studies were informed by an implementation framework or model. Huether and colleagues [36] utilised Cullens and Adams’ Implementation Strategies for Best Practice Guide. Jones and colleagues [33] used the Knowledge to Action (KTA) implementation framework; and Tian and colleagues [35] used the Promoting Action on Research Implementation in Health Services (PARIHS) framework to guide dissemination and implementation of CRF guidelines into clinical practice.

### Implementation strategies

Distinct EPOC implementation strategies used by all included studies are recorded in Table 1.

#### Educational meetings and materials

Coaching and training strategies were the most used strategies (five of six studies) [32–36] to promote uptake of CRF interventions. Health professional education was delivered in the form of regular staff meetings, staff training sessions and workshops, formal presentations, unit in-services, role-play sessions (i.e., participation in mock fatigue assessments & patient education sessions) and print materials (i.e., ‘pocket’ fatigue guidelines & tip sheets, flipcharts, newsletters, education booklets). Education content across each study varied but generally included background information on fatigue, fatigue management, fatigue assessment procedures and tools, and referral processes.

#### Local opinion leaders and stakeholder engagement

Stakeholder engagement were used in five of six studies [32, 33, 35–37]. Stakeholder groups commonly included clinicians (i.e., psychiatrists, radiation and medical oncologists, physical therapists, surgeons, professors, specialist nurses), cancer survivors, research staff (i.e., research assistants, postdoctoral fellows), and community support professionals. The use of a key opinion leader was described in only one study [35], engaging a nurse who trained and persuaded fellow clinicians to accept implementation efforts.

#### Use of clinical guidelines and local consensus processes

Three studies [36–38] focused efforts on implementing existing clinical fatigue guidelines, including the National Comprehensive Cancer Network (NCCN) Fatigue Guidelines [32, 34] and the Canadian Association of Psychosocial Oncology (CAPO) guidelines for CRF [33]. Tian and colleagues developed and implemented the Clinical Practice Guideline: Nursing Care of Cancer-Related Fatigue in Adults with Cancers [35], whereas Jones and colleagues specifically described the adaptation of guidelines to the local context after stakeholder consensus [33].

#### Audit and feedback

Specific audit and feedback strategies were described in two studies [32, 35]. One study [35] described periodic audit completion rounds on nursing units, and routine discussions with staff to gather concerns, challenges, suggestions and distribute feedback; however, study authors did not report the specific details of audit content. The second study [32] described health professional training; fatigue screening at patient admission; fatigue screening at regular intervals throughout care; delivery of comprehensive fatigue assessments; and the provision of management strategies (i.e., physical activity and other strategies) to patients as components that were audited. In addition, interviews with patients and reviewed patient records were used to measure clinician compliance with audit components. A third study did [36] report the use of audit and feedback strategies, however they did not specify processes in further detail.

#### Managerial supervision and managing performance

Managerial supervision and monitoring of performance strategies were utilised in two studies [32, 36] in the form of regular reporting to senior leaders, routine communication with nurses, and ongoing monitoring of staff to ensure program compliance. Wang and colleagues [32] reported that intervention leaders worked within the nursing unit to monitor nursing practice to ensure adequate nurse education on CRF, nurse provision of CRF assessment upon patient admission, and nurse delivery of patient education for CRF management strategies (i.e., physical activity, and other strategies). Unfortunately, Huether and colleagues [36] reported using managerial supervision and monitoring of performance strategies but did not provide specific detail on performance outcomes.

#### Continuous quality improvement

Continuous quality improvement strategies were described by three studies [35–37] and generally involved protocol revisions and program modification based on clinician or patient feedback. Of these studies, the ‘Energy Through Motion’ CRF program [36] reported the formation of a dedicated quality improvement program; however, details of this quality improvement program were not provided.

#### Tailored interventions

Four studies [32–35], described tailored interventions that were based on assessments of barriers to change. Wang and colleagues [32] conducted a clinical audit to determine CRF management barriers; Borneman and colleagues [34] identified barriers at the patient, professional and system level during the first phase of their quasi-experimental study; Tian and colleagues [35] identified barriers and facilitators through focus group discussions, surveys and observation; and Jones and colleagues [33] conducted semi-structured focus group interviews with stakeholder groups. Common barriers identified were lack of CRF knowledge, inconsistent application of CRF guidelines, insufficient knowledge of CRF screening and assessment, resistant attitudes towards program adoption, busy environments, heavy workloads, and time restrictions.

#### Reminders

Only one study [36] reported the regular use of practice reminders to reinforce the intervention to staff members. Reminders were distributed by the project leader through intervention ‘tips of the week’; however, the distribution method used (e.g., newsletter, text, email) remains unclear.

#### Organisational culture

Efforts to change organisational culture were reported across all studies [32–37] using practical methods including the formation of fatigue specific referral and clinical feedback systems; creation, and incorporation of CRF assessment flowcharts and assessment tools; addition of fatigue management processes to organisation protocol; and the development of CRF information documentation for both staff and patients.

### Implementation and intervention outcomes (RE-AIM)

Implementation outcomes of the included studies are outlined in Table and Additional File 4. Overall, Effectiveness and Implementation were the most highly reported dimensions followed by Reach. Adoption and Maintenance were the least reported dimensions.

### Reach of CRF interventions

Reach is defined as the number, proportion and representativeness of individuals who are willing to participate in a given initiative or intervention [31]. Descriptions of target population (including demographic information), inclusion criteria and sample size were reported in five [33–37] of six studies. Only one study [35] reported the representativeness or characteristics of participants and non-participants by comparing the sample with broader populations. Program participation rate was reported by two studies [33, 34].

### Efficacy of CRF interventions

Efficacy describes the impact of CRF interventions on identified outcomes (e.g., fatigue). Fatigue and behavioural outcome measures were reported in four of six studies [34–37]. Of these studies, all reported reduced CRF severity as a result of the intervention. The ‘Energy Through Motion’ CRF intervention [36] resulted in decreased fatigue severity by an average of two points compared to an increase of 0.69 points in the usual care arm (*p* = 0.0006). Pre- and post-program scores from Van Gerpen and Becker’s ‘LifeSpring’ CRF intervention [37] demonstrated statistically significant improvements in fatigue (5.58 (SD 2.11) vs. 3.55 (SD 1.86); *p* < 0.0001). Tian and colleagues’ [35] also produced lower patient CRF scores after their CRF intervention (*p* = 0.04). Lastly, the ‘Passport to Comfort’ CRF intervention [34] produced significant and beneficial effects on fatigue barriers (*p* = 0.001) and patient fatigue management knowledge (*p* = 0.002). No studies reported on cost-effectiveness*.*

### Adoption (setting and staff level) of CRF implementation efforts

Adoption is defined as the number, proportion, and representativeness of settings and intervention agents who are willing to initiate a program [31]. Indicators for adoption were the least reported outcomes in the included studies. Further, indicators such as the description of targeted locations, inclusion/exclusion criteria of settings and staff, method to identify settings and staff, setting and staff participation rate, representativeness of staff and settings, number of staff participating in intervention delivery, and measures of intervention cost were not reported by any study.

### Implementation

According to Glasgow and colleagues [31], implementation at the setting level refers to the cost of implementation, and whether the intervention was delivered as intended. At the individual level, implementation refers to clients’ use of the intervention and implementation strategies. Intervention completion rates were reported by four studies [32, 33, 36, 37] and ranged from 80 to 90%. No study described methods to ensure fidelity of the intervention. Additionally, only the ‘Energy Through Motion’ CRF program [36] detailed the ongoing implementation cost of the intervention (intervention kits valued at $21.75 USD per patient).

Results of implementation efforts varied across all studies. Implementation strategies utilised in Wang and colleagues’ study [32] resulted in increases in nurse CRF education, nurse assessment of patient CRF upon admission and at regular intervals throughout treatment, and nurse provision of patient education on exercise and other management strategies for CRF. Jones and colleagues [33] reported that their two-hour health and community professional training session resulted in large to very large increases in clinician CRF knowledge (d = 0.98), self-efficacy in CRF assessment (d = 0.88), self-efficacy to intervene for CRF (d = 1.13), and intent to apply CRF guidelines (d = 1.35). Tian and colleagues [35] dissemination of CRF guidelines led to increased clinician knowledge, attitude and CRF management behaviours, and the increased adoption of effective CRF management strategies amongst patients. Borneman and colleagues [36] strategies to address professional and system barriers (e.g., formal fatigue presentations to staff, monthly newsletters, ongoing meetings with nurse practitioners) resulted in organisational change (e.g., routine fatigue assessment added to outpatient clinic sheet, increased supportive care referrals). Although Huether and colleagues [36] and Van Gerpen and Becker [37] described the use of implementation strategies in their respective programs, outcomes of their implementation efforts were not reported.

### Maintenance of CRF interventions and implementation efforts

Maintenance is defined as the extent to which individual behaviour is sustained 6 months or more after the intervention; and whether a program or policy is institutionalised as part of routine organisational practice [31]. Maintenance indicators at both the individual and setting level were not fully reported in any study and only partially reported across four of the six studies [34–37]. Borneman and colleagues [34] reported individual follow-up and attrition (3 months) after program completion; however, did not provide follow-up data at ≥6 months post-intervention Tian and colleagues [35] stated that innovations and strategies developed from their implementation efforts were maintained for 2 months after project completion, but were discontinued due to lack of staff time and funding. Borneman and colleagues [34] noted their dissemination of the intervention was conducted at the study institution and that plans were underway to disseminate the intervention into other community centres.

At the time of writing this review, the ‘LifeSpring’ CRF intervention described in Van Gerpen and Becker’s evaluation [37] is currently maintained and institutionalised at the US Bryan Health Medical Center. Additionally, the ‘Energy Through Motion’ CRF intervention [36] appears to be institutionalised at survivorship clinics offered by the University of Iowa Holden Comprehensive Cancer Centre in the United States; however, little detail of the current program and its integration can be found. None of the studies provided details on costs associated with maintenance, however Van Gerpen and colleagues [37] state that ongoing funding for the program was provided by the medical centre’s foundation, and Huether and colleagues report that after project completion of the ‘Energy Through Motion’ CRF intervention, “continuation of funding for patient supplies was obtained through requests from a regular benefactor of the cancer centre” [36].

## Discussion

Efforts to sustainably implement evidenced-based CRF management strategies into routine clinical care are urgently needed, owing to the high incidence, prevalence, and burden of CRF in cancer survivors severely impacting health-related quality of life [1]. Despite established CRF management strategies [1, 16], sustainable models of care connecting cancer survivors to effective CRF interventions have yet to be satisfactorily investigated [39]. Our systematic scoping review was able to identify only six studies evaluating the implementation of interventions designed for individuals experiencing CRF. Further, all studies had limited external validity and lacked methodological rigor (e.g., poor reporting of exclusion criteria, study design, data analysis; limited to no follow-up periods; absence of frameworks and theories to guide implementation, etc).

Only three studies used specific implementation models to guide the dissemination process. When applied accurately, implementation theories and frameworks have been shown to enhance dissemination into practice by improving interpretability of study findings and increasing the use of essential implementation strategies [27]. Given only three studies adopted an implementation framework in our review, it is difficult to establish which model is the most helpful for future CRF implementation.

Despite limited use of implementation models, a range of strategies were used across included studies. While most studies in this review demonstrated immediate changes at the clinician, organisational and patient level through their use of various implementation strategies, it was difficult to determine the impact (and impact strength) of individual strategies on implementation outcomes. Further research to identify the preferred strategy from clinicians, patients, and other stakeholders in CRF interventions is likely to be helpful in ascertaining the usefulness, relevance, and effectiveness of specific implementations strategies that will improve implementation efforts [38].

Maintenance and Adoption were the least reported RE-AIM indicators, while Reach, Effectiveness, and Implementation were highly reported across the studies. For all domain indicators, reporting was exceptionally higher for aspects of internal validity (e.g., inclusion criteria, sample size) than external validity (e.g., representativeness of participants, description of settings and staff, intervention fidelity). This is consistent with previous reviews of health interventions across a variety of populations [30, 40–43]. Of note, the level of reporting on Adoption was poor with indicators at the staff and setting level amongst the lowest reported. Details of intervention settings and delivery staff are critical as they allow for the assessment of intervention applicability (and its effect) to different conditions [43]. In five of six included studies, intervention facilitators were employed solely to deliver the CRF intervention or implementation effort, and often had high levels of specific training and supervision, a situation which is not indicative of “real-life practice”. Thus, to assist the replication and translation of CRF guidelines and management interventions into routine practice; information regarding intervention setting and staff characteristics, and level of staff skill and training is vital.

Cost was another implementation outcome that was under-reported yet is essential when establishing sustainable models of care for cancer survivors. Cost effectiveness, including start-up and ongoing costs of intervention delivery, have been identified as key factors in determining the translation of research findings into practice [44]. However, these costs were rarely reported, with only the ‘Energy Through Motion’ CRF intervention providing an explicit cost of intervention materials ($21.75 USD per patient) [36]. Cost concerns are associated with reduced stakeholder willingness to implement evidenced-based interventions and represent the most significant barrier to evidenced-based practice implementation and program sustainability [45–47]. Reporting costs in future implementation efforts for CRF management is critical.

Assessment of intervention maintenance and sustainability has been identified as a neglected area in clinical research [30, 40–43, 48] with results of this review in agreement. Across all studies, the same common barriers to program continuation were reported: lack of clinician knowledge and skills in the management of CRF, shortage of clinician human resources, lack of program and staff funding, and lack of clinician time. These barriers have been repeatedly highlighted across the CRF literature [49–51]. Berger and Mooney [51] emphasise the lack of access to, and re-imbursement for, integrated supportive cancer programs and services remains the largest challenge to effectively implementing CRF guidelines into routine clinical practice. Further, they conclude that without additional time and reimbursement, clinicians cannot be expected to adequately provide effective or targeted clinical care to individuals experiencing CRF.

### Implications for future research and practice

Implementation research in CRF management is severely lacking, highlighting the need for focussed research in this area. In Table 2, we provide key findings and recommendations of our systematic scoping review. Although feasibility, acceptability and effectiveness outcomes are widely reported across CRF literature, a greater focus on other pertinent implementation outcomes such as adoption and program maintenance are paramount to translate CRF guidelines and interventions into real-world settings. While we acknowledge that these studies have different aims, and may not comprehensively cover all dimensions outlined in the RE-AIM framework, it is suggested that CRF implementation studies incorporate several stepwise iterative phases to provide opportunities to trial, assess and refine elements; determine resource needs and costs; and gather evidence of implementation impact [49].

**Table 2:** Key Findings and Implications for Future Research and Practice

**Future research should:**	
• put greater emphasis on reporting aspects of external validity such as representativeness, setting characteristics, staff level characteristics, and implementation cost.	
• be underpinned or guided by an implementation framework.	
• utilise rigorous pragmatic designs with adequately powered samples and longer follow-up periods.	
• report the impact of implementation at the system, health professional and cancer survivor level.	
**Intervention developers should:**	
• consider sources of ongoing funding and endeavour to use existing resources (staff, equipment, infrastructure, etc.) to deliver implementation efforts.	
**Clinical leaders should:**	
• endeavour to build clinician awareness and knowledge of evidenced-based CRF management and assessment strategies through the provision of educational training and resources.	
• seek regular engagement with clinical staff and relevant stakeholder groups to identify potential/existing enablers or barriers to clinical change and to tailor implementation efforts to specific contexts.	
• place emphasis on the allocation of clinician resources within settings, the provision of time management support to clinicians (e.g., reallocation of work tasks, adjustment of procedures to fit clinician schedule, adjustment of clinician schedule to fit procedures) and the identification of ‘clinical champions’ or opinion leaders, to encourage peer behaviour change and compliance with the recommended CRF management and assessment practices.	

Most studies included in this review described CRF implementation at the health professional level, or in acute health care settings. However, the physical, psychological, and psychosocial needs of cancer survivors after active treatment require continuous long-term support which is often only provided by primary and community health care teams [52]. As such, there is a need to extend CRF implementation and translation efforts to community and primary care settings where they will be more accessible to larger populations of cancer survivors in the community.

### Limitations

This review has two main limitations. First, the inconsistencies of what constitutes an implementation study (e.g., implementation processes, terminologies, definitions, intention to treat, inclusion/exclusion criteria) made it difficult to detect a distinct relationship between the use of implementation models and strategies, and implementation outcomes. However, this is not unique to our review with similar inconsistencies frequently reported across the implementation science literature [53–55]. Second, our review was limited to studies in English, potentially resulting in some level of publication bias limiting the generalisability of results.

## Conclusion

This systematic scoping review is the first to examine models, strategies, and outcomes of studies reporting on the implementation of interventions for individuals experiencing cancer-related fatigue. Our review found that various implementation strategies have been used to promote uptake of CRF management interventions and guidelines at the organisational, clinician, and patient level. However, lack of consistent reporting of external indicators (e.g., ongoing and start-up costs of intervention, setting and staff representativeness) and factors such as lack of clinician time, insufficient clinician and intervention funding, and unsustainable maintenance costs, are potential barriers to study translatability and CRF program implementation. This review emphasises the absence of quality CRF implementation studies and highlights the pertinent need for more robust, theory driven implementation studies to bridge this important knowledge-practice gap.

## Supplementary Information

**Additional file 1.** Preferred Reporting Items for Systematic reviews and Meta-Analyses extension for Scoping Reviews (PRISMA-ScR) Checklist.

**Additional file 2.** Modified EPOC Implementation Strategies.

**Additional file 3.** Search Strategy.

**Additional file 4.** RE-AIM Indicators & Reporting of RE-AIM Indicators Across all Included Studies.

## Data Availability

Data sharing is not applicable to this article as no datasets were generated or analysed during the current study.

## Abbreviations

CAPO: Canadian Association of Psychosocial Oncology
CRF: Cancer-related fatigue
EPOC: Effective Practice and Organisation of Care Group
KTA: Knowledge to Action Implementation Framework
NCCN: National Comprehensive Cancer Network
PARIHS: Promoting Action on Research Implementation in Health Services Framework.
PRISMA-ScR: Preferred Reporting Items for Systematic Reviews and Meta-Analyses Extension for Scoping Reviews
QOL: Quality of Life
RE-AIM: Reach Effectiveness Adoption Implementation Maintenance
WES: Weighted effect size
