# Correction to: Bridging the research to practice gap: a systematic scoping review of implementation of interventions for cancer-related fatigue management

**DOI:** 10.1186/s12885-021-08619-5

**Published:** 2021-08-02

**Authors:** Oluwaseyifunmi Andi Agbejule, Nicolas H. Hart, Stuart Ekberg, Bogda Koczwara, Rahul Ladwa, Camilla Simonsen, Elizabeth P. Pinkham, Raymond Javan Chan

**Affiliations:** 1 Cancer and Palliative Care Outcomes Centre, School of Nursing, Queensland University of Technology (QUT), N Block, Kelvin Grove Campus, Kelvin Grove, Queensland 4059 Australia; 2 Exercise Medicine Research Institute, Edith Cowan University, Perth, Western Australia 6027 Australia; 3 Institute for Health Research, University of Notre Dame Australia, Fremantle, Western Australia 6959 Australia; 4 Flinders University and Flinders Medical Centre, Flinders Drive, Bedford Park, South Australia 5048 Australia; 5 Princess Alexandra Hospital, Metro South Hospital and Health Services, Woolloongabba, Queensland 4102 Australia; 6 School of Medicine, University of Queensland, St Lucia, Queensland 4072 Australia; 7 School of Health and Behavioural Science, University of Queensland, St Lucia, Queensland 4072 Australia

**Correction to: BMC Cancer 21, 809 (2021)**

**https://doi.org/10.1186/s12885-021-08394-3**

Following publication of the original article [1], the authors identified an error in the author name of Bogda Koczwara. The given name and family name were erroneously transposed.

The incorrect author name is: Koczwara Bogda

The correct author name is: Bogda Koczwara

The author group has been updated above and the original article [1] has been corrected.
