# Supplementary material for: Bridging the research to practice gap: a systematic scoping review of implementation of interventions for cancer-related fatigue management
Source: BMC Cancer. 2021 Jul 14;21:809. doi: 10.1186/s12885-021-08394-3 (PMC8278687; doi:10.1186/s12885-021-08394-3)
Supplement: Supplementary file 2 — Additional file 2. Modified EPOC Implementation Strategies. [file 12885_2021_8394_MOESM2_ESM.docx]

| **Additional File 2:** Modified EPOC Implementation Strategies | |
| --- | --- |
| STRATEGY | CRITERIA |
| Organisational culture | Did the intervention utilise any strategies to change organisational culture? |
| Audit and Feedback | Is performance feedback provided in either a verbal, written or electronic format? Are recommendations for clinical action provided? |
| Communities of practice | Does the intervention utilise communities in practice?  (Groups of people with a common interest who deepen their knowledge and expertise in an area by interacting on an ongoing basis) |
| Continuous Quality Improvement | Does the article or intervention describe/undertake any iterative processes to review and improve care? (This can include the involvement of healthcare teams, analysis of a process or system, a structured process improvement method or problem-solving approach, and use of data analysis to assess changes). |
| Monitoring the performance of the delivery of healthcare | Is the intervention/program/ service routinely monitored. Has it been compared with an external standard? |
| Educational games | Does the intervention use games as an educational strategy to improve standards of care |
| Educational materials | Does the article or intervention describe/ incorporate the distribution of knowledge and educational materials to support clinical care?  For example, this may be facilitated by the internet, learning critical appraisal skills; skills for electronic retrieval of information, diagnostic formulation; question formulation, etc. |
| Educational meetings | Does the intervention or article describe or use courses, workshops, conferences or other educational meetings? |
| Educational outreach visit, or academic detailing | Does the article/ intervention describe or utilise personal visits by a trained person to facilitators/intervention participants/ in their own settings (to provide information with the aim of changing practice). |
| Clinical Practice Guidelines/ Local Consensus Processes | Is the development of the intervention informed by any clinical practice guidelines, frameworks or models? Does the article promote the implementation of clinical guidelines? Was there any formal or informal consensus process to choose or adapt guidelines for use? |
| Inter-professional Education | Does the intervention use/provide/incorporate continued education of health professionals? |
| Local opinion leaders | Were local opinion leaders identified and used to inform intervention development and to promote good clinical practice? |
| Patient-mediated interventions | Was the intervention informed by information provided by patients (survey, interview, other patient interactions)? |
| Managerial supervision | Does the article/ intervention incorporate routine supervision visits with health? |
| Routine patient reported outcomes | Does the article or intervention describe/allow for the facilitation of frequent routine patient reported outcome measures (e.g., logbook recording fatigue severity, impact on QOL, etc.)? |
| Reminders | Does the intervention use or describe any manual or computerised mechanisms that prompt individuals to perform an action? |
| Tailored interventions | Is the intervention based off any assessment of barriers to change? Was the intervention informed by any assessments like interviews or surveys? |

Summary of EPOC Modifications

- Implementation strategies put into question format to assist screening process.
- EPOC taxonomy contains more items. Only the taxonomy relevant to the study were included (see summary of EPOC modifications)

The following strategies were not included as they were not considered relevant to the study (outside the scope of the study or a different scope of the study): Public release of performance data, Clinical incident reporting and the “Interventions targeted at specific types of practice, conditions or settings” section.
