# Supplementary material for: Bridging the research to practice gap: a systematic scoping review of implementation of interventions for cancer-related fatigue management
Source: BMC Cancer. 2021 Jul 14;21:809. doi: 10.1186/s12885-021-08394-3 (PMC8278687; doi:10.1186/s12885-021-08394-3)
Supplement: Supplementary file 3 — Additional file 3. Search Strategy. [file 12885_2021_8394_MOESM3_ESM.docx]

**Additional File 3: Search Strategy**

CINAHL

1. (MH "Systems Implementation") OR (MH "Program Implementation") OR (MH "Implementation Science") OR (MH "Program Development") [53,225]
2. TI ( implement* OR disseminat* OR translat* OR ("Systems Implementation") OR ("Program Implementation") OR ("Implementation Science") OR (Program Development) ) OR AB ( implement* OR disseminat* OR translat* OR ("Systems Implementation") OR ("Program Implementation") OR ("Implementation Science") OR ("Program Development") ) [266,712]
3. S1 OR S2 [299,074]
4. (MM "Cancer Fatigue") [959]
5. (MH "Cancer Fatigue") [1,590]
6. TI ( ("cancer fatigue" OR "cancer related fatigue" OR cancer related fatigue) ) OR AB ( ("cancer fatigue" OR "cancer related fatigue" OR cancer related fatigue) ) [1,380]
7. S4 OR S5 OR S6 [2,435]
8. (S3 AND S7) **[159]**

EMBASE

1. 'implementation science'/exp [1510]
2. implement*:ab,ti OR translat*:ab,ti OR 'program development':ab,ti [1,060,964]
3. #1 OR #2 1,061,142]
4. 'cancer fatigue'/exp/mj [1,226]
5. 'cancer fatigue'/exp [3,179]
6. 'cancer fatigue':ab,ti OR 'cancer related fatigue':ab,ti [2,270]
7. #4 OR #5 OR #6 [4,395]
8. #3 AND #7 **[251]**

COCHRANE LIBRARY

1. MeSH descriptor: [Implementation Science] explode all trees [34]
2. MeSH descriptor: [Health Plan Implementation] explode all trees [176]
3. MeSH descriptor: [Translational Medical Research] explode all trees [116]
4. MeSH descriptor: [Program Development] explode all trees [710]
5. (implement* OR translation OR "program development"):ti,ab,kw [42,849]
6. ("cancer related fatigue" OR "cancer fatigue"):ti,ab,kw [1,016]
7. (#1 OR #2 OR #3 OR #5) AND #6 **[52]**

PUBMED: (((("implementation science"[MeSH Terms] OR "translational medical research"[MeSH Terms] OR "program development"[MeSH Terms] OR knowledge translation[MeSH Terms] OR health plan implementation[MeSH Terms] OR (implement*[Title/Abstract]) OR (translation[Title/Abstract])))) AND ((("cancer fatigue"[Title/Abstract]) OR "cancer related fatigue"[Title/Abstract])) **[99]**
