# Supplementary material for: Bridging the research to practice gap: a systematic scoping review of implementation of interventions for cancer-related fatigue management
Source: BMC Cancer. 2021 Jul 14;21:809. doi: 10.1186/s12885-021-08394-3 (PMC8278687; doi:10.1186/s12885-021-08394-3)
Supplement: Supplementary file 4 — Additional file 4. RE-AIM Indicators & Reporting of RE-AIM Indicators Across all Included Studies. [file 12885_2021_8394_MOESM4_ESM.docx]

**Additional File 4:** RE-AIM Indicators & Reporting of RE-AIM Indicators Across all Included Studies

| REACH |  |
| --- | --- |
| R1. Described Target Population & Demographics | A brief description of the broader target population & Gender, age, educational attainment, occupation, SES, behavioural  outcomes |
| R2. Method to Identify Target Population | Describe the process by which the target population was identified for  participation in the study. |
| R3. Recruitment Strategies | Describe the methods used to recruit participants into the study. |
| R4. Inclusion Criteria | Description of inclusion criteria for individual participants |
| R5. Exclusion Criteria | Description of exclusion criteria for individual participants |
| R6. Representativeness of participants | Comparison of characteristics (% females, mean age, age range...) between participants and non-participants |
| R7. # Eligible & Invited (exposed) to Recruitment | The total number of eligible participants contacted for participation. |
| R8. Sample Size | The number of people who agree to participate |
| R9. Participation Rate | Sample size divided by the target population denominator |
| R10. Cost of Recruitment | The cost of recruitment can reflect monetary and/or time units. |
| EFFICACY / EFFECTIVENESS |  |
| E1. Measures and results for post-intervention assessment | Description of outcome measures |
| E2. Intention-to-treat analysis utilised | Analysing participants in the groups in which they were randomised regardless of adherence |
| E3. Imputation procedure | Description of imputation procedure used for Intention-to-treat analysis |
| E4. Quality of Life measure included | Description of use and results for QoL |
| E5. Measure of satisfaction with / acceptability of programme | Description of included measures regarding satisfaction and acceptability |
| E6. Cost Effectiveness | Code as reported if specific mention and amounts are provided for the cost of the intervention. |
| E7. Effects at follow-up | At least one follow-up assessment (after post-intervention assessment) included |
| E8. Attrition at program completion | Description of type and % of attrition |
| ADOPTION |  |
| A1. Setting Adoption rate | The proportion of sites eligible and contacted that participated. |
| A2. Description of Targeted Location | Characteristics that would be considered an ideal location for the intervention. |
| A3. Inclusion/Exclusion Criteria of Setting | Description of eligibility criteria for settings |
| A4. Description of Intervention Location | The explicit statement of characteristics of the location of the intervention |
| A5. Method to Identify Setting | Describe the process by which the location was identified for participation in the study |
| A6. Average # of Persons Served Per Setting | Calculated average number of participants at each site. |
| A7. Staff Participation Rate | The proportion of the staff that was eligible and contacted and participated |
| A8. Method to Identify Target Delivery Agent | Describe the process by which the target delivery agent was identified for participation in the study. |
| A9. Description of Delivery Agent | Characteristics of Staff |
| A10.Level of Expertise of Delivery Agent | Description of expertise (e.g. training) of delivery agents |
| A11. Inclusion/Exclusion Criteria of Delivery Agent | The explicit statement of characteristics of the delivery agents that were used to determine if a potential delivery agent is eligible to participate. |
| A12. Measures of Cost Adoption | Description of costs for intervention set-up |
| IMPLEMENTATION |  |
| I1. Theories | Explicit statement of theories or principles used to develop the intervention |
| I2. Intervention Number of Contacts | Total number of encounters with participants |
| I3. Timing of Contacts | Describe when the intervention contacts occur over the course of the intervention. |
| I4. Duration of contacts | Length of each intervention contact |
| I5. Extent to which intervention was delivered as intended | Description of individual adherence, compliance, attendance and/or staff adherence |
| I6. Consistency of intervention delivery | Description of consistency across staff, time, settings and subgroups of participants |
| I7. Participant Attendance/ Completion rates | The proportion of the intervention that the participants received, on average. |
| I8. Costs of delivery (ongoing costs) | Description of costs (time, money) |
| MAINTENANCE |  |
| M1. Assessed outcomes ≥ 6 months | Follow-up periods ≥ 6 months post-intervention |
| M2. Drop-out rate to last follow-up | % drop-out (if follow-up assessment ≥ 6 months) |
| M3. Current status of programme | Description of current status after end of study |
| M4. Use of qualitative methods to measure individual or organisational level maintenance |  |
| M5. Is the program still in place | Description of program continuation after completion of the research  study. |
| M6. Adaptations made | Description of adaptations made to foster sustainability of programme |
| M7. Costs of maintenance | Description of costs (money, time) related to sustainability of programme |

1. Harden SM, Gaglio B, Shoup JA, Kinney KA, Johnson SB, Brito F, Blackman KC, Zoellner JM, Hill JL, Almeida FA *et al*: **Fidelity to and comparative results across behavioral interventions evaluated through the RE-AIM framework: a systematic review**. *Syst Rev* 2015, **4**:155.

Reporting of RE-AIM Indicators Across all Included Studies

|  | **REACH** | | | | | | | | | | **EFFECTIVENESS** | | | | | | | **ADOPTION** | | | | | | | | | | | | **IMPLEMENTATION** | | | | | | | | **MAINTENANCE** | | | | | | |
| --- | --- | --- | --- | --- | --- | --- | --- | --- | --- | --- | --- | --- | --- | --- | --- | --- | --- | --- | --- | --- | --- | --- | --- | --- | --- | --- | --- | --- | --- | --- | --- | --- | --- | --- | --- | --- | --- | --- | --- | --- | --- | --- | --- | --- |
|  | R1 | R2 | R3 | R4 | R5 | R6 | R7 | R8 | R9 | R10 | E1 | E2 | E3 | E4 | E5 | E6 | E7 | A1 | A2 | A3 | A4 | A5 | A6 | A7 | A8 | A9 | A10 | A11 | A12 | I1 | I2 | I3 | I4 | I5 | I6 | I7 | I8 | M1 | M2 | M3 | M4 | M5 | M6 | M7 |
| Abbott et al. 2017 & Huether et al., 2016 | + | - | + | + | + | - | - | + | - | - | + | - | - | + | + | - | + | + | - | - | + | - | - | - | - | + | + | - | - | + | + | + | + | - | + | + | + | - | - | + | - | Y | - | - |
| Borneman et al., 2010 & 2011 | + | - | - | + | - | - | -,+ | + | -,+ | - | + | - | + | + | - | - | +,- | - | - | - | + | - | + | - | - | + | + | - | - | - | + | **+** | + | - | - | - | - | - | - | + | - | - | - | - |
| Jones et al., 2020 | + | - | - | + | - | - | - | + | + | - | - | - | - | - | - | - | - | - | - | - | - | - | - | - | - | + | + | - | - | + | + | + | + | - | - | + | - | - | - | - | - | - | - | - |
| Tian et. al 2017 | + | - | - | + | - | - | - | + | - | - | + | - | - | - | - | - | - | - | - | - | + | - | + | - | - | - | - | - | - | + | - | - | - | - | - | - | - | - | - | + | - | - | - | - |
| Van Gerpen & Becker, 2013 | + | - | + | + | - | - | - | + | - | - | + | - | - | + | + | - | - | - | - | - | - | - | + | - | - | + | + | - | - | - | + | + | + | - | - | + | - | - | - | + | - | Y | + | - |
| Wang et al., 2018 | - | - | - | - | - | - | - | - | - | - | - | - | - | - | - | - | - | - | - | - | + | - | - | - | - | - | - | - | - | - | + | - | - | - | - | + | - | - | - | - | - | - | - | - |

(N.A) Not Applicable, (+) reported, (-) not-reported, (Y) yes, (N) no.
